# Evaluation of PKM2 and MAPK8IP2 Polymorphism in Ameloblastic Carcinoma: A Retrospective Quantitative Study

**Published:** 2012

**Authors:** Abbas Khodayari, Seyyed Mohammad Hossein Ghaderian, Mohammad Jafarian, Alireza Jahangirnia, Alireza Nayebi, Fahimeh Akhlaghi, Nasim Taghavi, Reza Akbarzadeh Najar, Sanaz Tabarestani, Arash Khojasteh, Sarah Aghabozorg Afjeh

**Affiliations:** 1 *Department of Oral and Maxillofacial Surgery, Dental Research Center, Shahid Beheshti University of Medical Sciences, Tehran, Iran.*; 2 *Department of Medical Genetics, Shahid Beheshti University of Medical Sciences, Tehran, Iran.*; 3 *Department of Oral and Maxillofacial Surgery, Shahid Beheshti University of Medical Sciences, Tehran, Iran.*; 4 *Department of Oral and Maxillofacial Surgery, Shahid Beheshti University of Medical Sciences, Tehran, Iran.*; 5 *Department of oral and maxillofacial pathology, dental faculty, Shahid Beheshti university of medical science, Tehran,Iran.*

**Keywords:** Ameloblastic carcinoma, PKM2, MAPK8IP2, polymorphism

## Abstract

Ameloblastic carcinoma (AC) is a rare malignant epithelial odontogenic tumor that histologically retains the features of ameloblastic differentiation and exhibits cytological features of malignancy in the primary or recurrent tumor. It may develop within a preexisting ameloblastoma or arise de novo or from an odontogenic cyst. Epidemiological evidence shows that human cancer is generally caused by genotoxic factors, genes involved in the susceptibility of cancer, including those involved in metabolism or detoxification of genotoxic environment and those controlling DNA replication. Nowadays, gene polymorphism has an important role in development of malignant tumor. We report a case series study of ameloblastic carcinoma and ameloblastoma to show the role of PKM2 and MAPK8IP2 polymorphisms in these tumors. The DNA was extracted separately from specimens in paraffin sections of the tumor. Polymorphism of these genes was determined by PCR-RFLP (Polymerase Chain Reaction-Restriction fragment length polymorphism) method. The allele distributions of all samples were in Hardy-Weinberg equilibrium. The genotype and allele distribution in these genes were not statistically different between patients and controls.

Ameloblastomas are usually benign, locally aggressive neoplasms derived from the epithelial odontogenic tissues, which are part of the tooth-forming apparatus (1). Malignant tumors of ameloblastic origin are very rare, and appear in two different scenarios: malignant ameloblastoma, defined as a well-differentiated cytologicaly benign odontogenic tumor that paradoxically metastasizes, and ameloblastic carcinoma which refers to cytologically malignant tumors that may or may not metastasize (2, 3).

Ameloblastic carcinoma (AC) is a rare malignant epithelial odontogenic tumor that histologically retains the features of ameloblastic differentiation and exhibits cytological features of malignancy in the primary or recurrent tumor. It may develop within a preexisting ameloblastoma or arise de novo or from an odontogenic cyst (4-6).

Due to the relatively high incidence of ameloblastoma and rarity of ameloblastic carcinoma, the lack of a specific medical criteria to differentiate the two from each other, and the malignancies side effects for patients, finding effective genetic and immunohistochemical factors for this malignancy, is considered as a great advance in prevention and treatment of the disease (7). Many researchers believe that the accumulation of multiple genetic defects is necessary for representing malignant phenotypes (8).

Clinical studies have shown that individuals who are at significantly increased genetic risk of ameloblastic carcinoma can be identified through cancer predisposition genetic testing. The ability to identify high-risk individuals who may benefit from cancer prevention and early cancer detection strategies can improve their length and quality of life. On the other hand, the lack of clinical methods for identifying individuals with high risk of oral cancer, and to answer the question in the case of different responses of individuals to environmental carcinogens and differences in genetic predisposition, make importance of using genetic tests for ameloblastic carcinoma more under-standable (9, 10).

During the last few decades, extensive effort has been invested in identifying sources of genetic susceptibility to cancer. Both the International Human Genome Sequencing Project and the International HapMap Project have generated a very large amount of data on the location, quantity, type, and frequency of genetic variants in the human genome. Facilitated by continuing technological advances that allow faster and cheaper genotyping results, a large and increasing number of observational studies investigating the association between variants in candidate genes and cancer risk have emerged (11). Nowadays, gene polymorphism has an important role in activating metabolizing enzymes and development of malignant tumor (Genetic polymorphism is defined as variations in genome sequences that may be beneficial or harmful).

Epidemiological evidence shows that human cancer is generally caused by genotoxic factors, genes involved in the susceptibility of cancer, including those involved in metabolism or detoxification of genotoxic environment and those controlling DNA replication (12). Although the molecular and genetic characteristics of ameloblastoma are still poorly understood, the cloning and characterization of expression of the ameloblastin (AMBN) and amelogenin genes in these tumors supports the hypothesis that ameloblastomas arise from the dental lamina, the outer enamel epithelium or the inner enamel epithelium (13-15).

Studies show that mutations of multiple genes in different pathways play an important role in malignancies. In some researches a decrease of expression of some genes, PKM2 and MAPK8IP2, and hypermethylation of P53 in AC has been reported (16, 17). So, it is likely that their lack of activity is involved in the process of tumor angiogenesis. PKM2 gene (Pyruvate kinase, muscle 2) is located on chromosome 15 (15q22). This gene encodes a protein involved in glycolysis. The encoded protein is a pyruvate kinase that catalyzes the transfer of a phosphoryl group from phosphoenolpyruvate to ADP, generating ATP and pyruvate.

MAPK8IP2 gene (mitogen-activated protein kinase 8 interacting protein 2) is located on chromosome 22 (22q13.33). The protein encoded by this gene is closely related to MAPK8IP1/IB1/JIP-1, a scaffold protein that is involved in the c-Jun amino-terminal kinase signaling pathway. This pathway plays a role in inflammatory signal transduction.

With the aim of finding new cancer genetic predisposition factors, we selected these two genes and evaluated their polymorphism in ameloblastic carcinoma. According to a variety of amloblastoma tumor and amloblastic carcinoma in terms of pathology as well as the symptoms of this tumor, it seems that the cellular and molecular processes which contribute to its division could offer early detection along with exclusive treatments strategy (18-20). 

## Materials and Methods

Clinical and histologic information from available documents in the archives of the Department of Oral and Maxillofacial Pathology, Taleghani Hospital and the Department of Oral and Maxillofacial Pathology, Shahid Beheshti University of Medical Sciences were reviewed. The Iranian Center for Dental Research approved all scientific and ethical issues for this study .We investigated 18 samples consisting of 6 normal follicles as controls, 6 samples of ameloblastoma and 6 samples of ameloblastic carcinoma .The case series study of ameloblastic carcinoma and ameloblastoma was identified by clinical and pathological findings. In these cases we also investigated c.37G>A polymorphism in PKM2 and the c.196C>T polymorphism in MAPK8IP2 polymorphism in these tumors. The DNA was extracted separately from specimens in paraffin sections of the tumor. Polymorphism of these genes was determined by PCR-RFLP (Polymerase Chain Reaction - Restriction fragment length polymorphism) method (21).

PKM2 genotyping was carried out according to Dall’Olio et al. method [6] based on the PKM2 gene GenBank: AM182983.2 and AM182984.2 sequences. The identified mutation was the transition G>A in the position of 37. The gene sequence of the length 231 bp was amplified. The thermal profile of PCR reaction was following: initial denaturation at 95°C for 5 minutes, 35 cycles for 30 s at 95°C, 30 s at 65°C and 30 s at 72°C and the final extension at 72°C for 5 minutes. The obtained DNA sequence was subject to digestion with restrictive enzyme ApoI (Fermentas) for 3.5 h at 37°C (21). To evaluate c.196C>T polymorphism in MAPK8IP2 gene, appropriate restriction enzyme, EcoRI, was used. The gene sequence of the length 210bp was amplified. Primers sequences used for detection of MAPK8IP2 mutations were 5'GCCAGCATCCTCTGAGTACC3' (Forward) and 5'GTGGGCTGTTTCCTCTGG3' (Reverse) (22).

## Results

**Fig 1:**
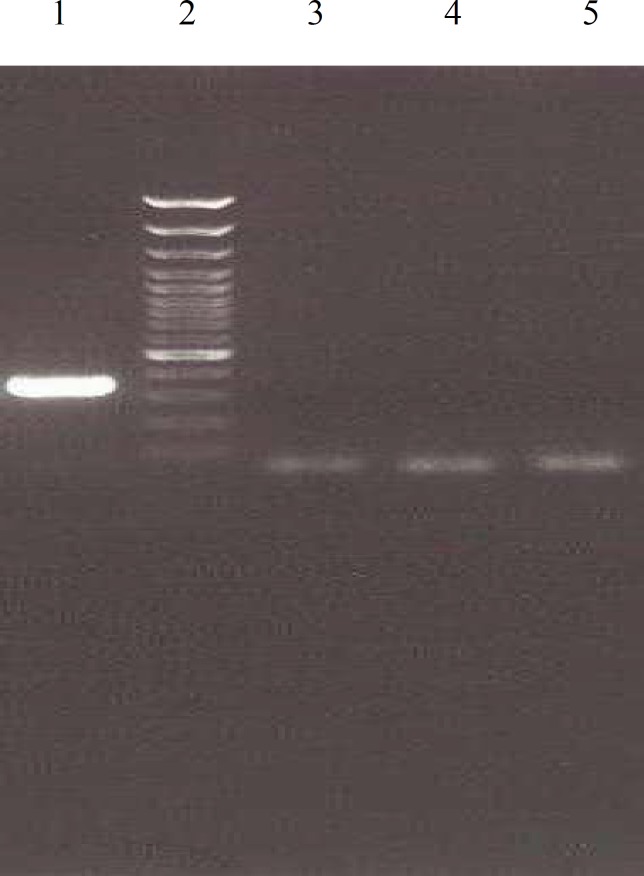
Gel electrophoresis for evaluation c.37G>A polymorphism in PKM2 gene. Lane 1: Undigested PCR product (231 bp); Lane 2: .molecular weight marker (50bp); Lane 3: wisdom tooth follicle; Lane 4: Ameloblastoma; Lane 5: Ameloblastic Carcinoma. There isnt any band for c.37G>A polymorphism in PKM2 gene in in the 3 studied samples

**Fig 2:**
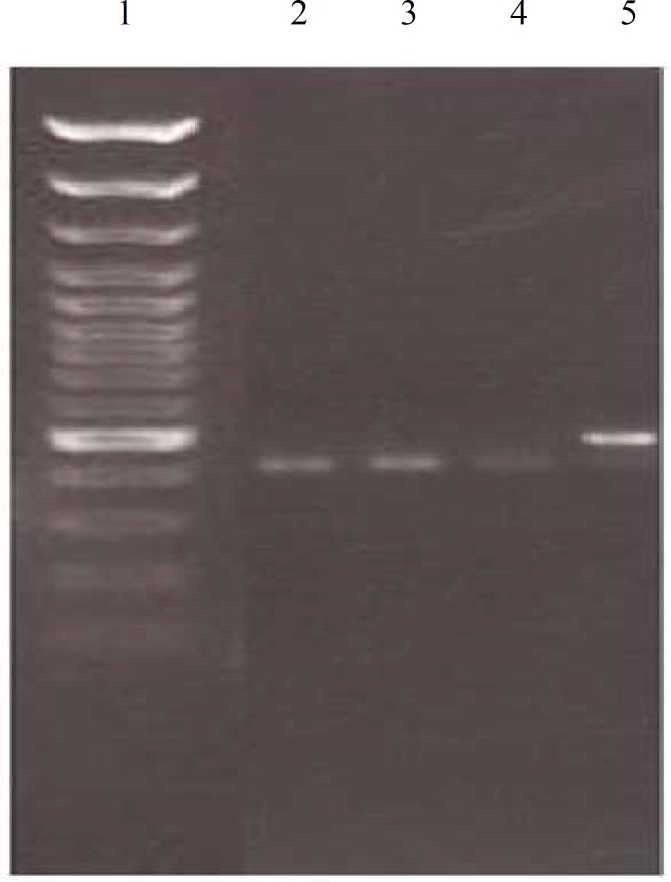
Gel electrophoresis for evaluation c.196C>T polymorphism in MAPK8IP2 gene. Lane 1: molecular weight marker (50bp); Lane 2: wisdom tooth follicle; Lane 3: Ameloblastoma; Lane 4: Ameloblastic Carcinoma; Lane 5: Undigested PCR product (210bp). There isnt any bad for c.196C>T polymorphism in the 3 studied samples

To assess whether PKM2 and MAPK8IP2 polymorphisms are associated with ameloblastic carcinoma, the patients with ameloblastic carcinoma were selected and compared to control individuals. The allele distributions of all samples were in Hardy–Weinberg equilibrium. The genotype and allele distribution in these genes were not statistically different between patients and controls. The presence of PKM2 and MAPK8IP2 gene expression were determined by PCR in ameloblastoma and ameloblastic carcinoma com-paring with normal follicle (controls).c.37G>A polymorphism in PKM2 and the c.196C>T polymorphism in MAPK8IP2 were not recognized in all of the sample cases. Figures 1 and 2 represent the electrophoretic pattern of PCR-RFLP evaluation for PKM2 and MAPK8IP2 respectively.

## Discussion

The rare and malignant type of ameloblastoma was mentiond in 1950 in oral pathology book of Dr. Thoma for the first time (18). 

Based on the 1971 WHO histological classification, Ameloblastoma is a benign Odonto-genic tumor (OTs) that is derived from epithelial, ectomesenchymal and/or mesenchymal elements of the tooth-forming apparatus. On the other hand, regardless of metastatic status, ameloblastic carcinoma is considered as a malignant neoplasm (23). However based on Willis descriptions, both lesions are malignant neoplasms which are characterized by slow growth and local aggressive specifications (24). In 2005, Who described ameloblastoma as a benign tumor again and not a malignant lesion (25).

According to our comprehensive studies, Dr. Carcinci's study was the only and the first specific study on ameloblastic carcinomas affected genes (18). That was a case-control study and the report was based on only one sample. Due to freshness of the sample, RNA was extracted and cDNA was synthesized. According to Dr. Carcinci's article and due to PKM2 and MAPK8IP2 genes mutations report in esophagus, breast and kidney cancers (24, 26, 27) and availability of the genes studying's kit, genetic study for evaluation of PKM2 and MAPK8IP2 polymorphisms in Ameloblastic carcinoma was performed in our study. In 2004, Koss performed study on the role of PKM 2 and its expression in esophagus adenocarcinoma and demonstrated its over expression (27).

In 2008, Shimada proposed the effect of two PKM2 gene and PML tumor suppressor protein breast cancer. Results of the study showed that down-regulation of PKM2 under the effect of PML and its loss of function causes cancer (28). 

We didn’t find c.37G>A polymorphism and the c.196C>T polymorphism in MAPK8IP2 in our samples. Studies show that mutation isn’t the only cause of cancer and other changes as alteration in protein synthesizing stages may playarole in cancer. For example defects in post modification of protein can be the cause of cancer. So, although Down-Regulation and Up-Regulation of genes are mentioned in Carcinci study as cause of Amelo-blastic carcinoma, but our results (not finding any mutation or alteration in the exon) can't prove lack of mutations, and on the other hand, other polymorphisms, matter of our population and defects in post modification of protein can be related to our negative results. 

The techniques and the size of samples may also be changed to improve these investigations. As the PCR-RFLP method has been used in this study, we cannot exclude consequently any nucleotide changes outside the recognition sequences of the restriction enzymes used, in the corresponding genes. Therefore, we suggest sequencing of those genes to find any SNPs or other changes in the genes.
